# Factor XI and Cancer: Physiopathological Linkage and Clinical Perspectives

**DOI:** 10.3390/jcm14176341

**Published:** 2025-09-08

**Authors:** Alfredo Mauriello, Anna Chiara Maratea, Celeste Fonderico, Vincenzo Quagliariello, Fabrizio Maurea, Nicola Maurea

**Affiliations:** 1 Division of Cardiology, Istituto Nazionale Tumori-IRCCS-Fondazione G. Pascale, Via M. Semmola, 52, 80131 Naples, Italy; annachiara.maratea@gmail.com (A.C.M.); celeste.fonderico@istitutotumori.na.it (C.F.); quagliariello.enzo@gmail.com (V.Q.); n.maurea@istitutotumori.na.it (N.M.); 2 Department of Advanced Biomedical Sciences, Federico II University of Naples, Via Pansini, 5, 80131 Naples, Italy; fabrizio.maurea@studenti.unina.it

**Keywords:** cancer, thromboembolism, bleeding, factor XI, anticoagulant, factor XI inhibitors

## Abstract

Thrombotic complications are a common cause of morbidity and mortality in cancer patients. Factor XI (FXI) appears to play a direct role not only in thrombotic pathogenesis but also in cancer progression. This comprehensive review aims to define the pathophysiological relationships between FXI and cancer and to assess existing therapeutic opportunities targeting this factor. This review highlights how FXI is implicated in tumor growth, tumor cell adhesion and migration, inflammation, and angiogenesis. FXI inhibition has been shown to reduce the risk of thrombosis, with a potentially improved safety profile in terms of bleeding risk. Several molecules, such as asundexian and abelacimab, are in clinical trials for the prevention and treatment of venous thromboembolic events, catheter-related thrombosis, and arterial thromboembolic events in cancer patients. In conclusion, factor XI is closely linked to the pathogenesis of cancer and its thromboembolic complications. The use of FXI inhibitors emerges as a promising therapeutic strategy, offering potentially positive effects in the prevention and treatment of thromboembolic complications without significantly increasing the risk of bleeding, a limitation of conventional anticoagulants. The preliminary evidence is that further clinical trials are required and that the available data is not enough to make firm clinical recommendations.

## 1. Introduction

Venous thromboembolism (VTE) includes pulmonary embolism (PE) and deep-venous thrombosis (DVT) [1]. Cancer-associated thrombosis (CAT) also encompasses manifestations of venous thrombosis or PE occurring during cancer evolution [2]. The lifetime risk of VTE is estimated to be 8% overall among general adults [3]; it is 4–7-fold higher in cancer patients [4,5,6], with varying incidences related to the type of cancer (the highest risk is for stomach, pancreatic, ovaric, and lung cancer and multiple myeloma) as well as the stage of cancer itself (metastatic/local advanced disease is at the highest risk) [4,5,6,7,8]. VTE is the second cause of death after cancer progression (>60% within the first year following VTE diagnosis) [5,6,7]. In addition, patients with cancer have a high risk of recurrence (up to 20% in the first 12 months after anticoagulation interruption), with a case fatality rate of almost 15%, even during therapeutic anticoagulation [5,6,7].

The relationship between coagulation and cancer is a complex and bidirectional interaction. Cancer promotes a state of hypercoagulability, known as Trousseau syndrome, which increases the risk of thromboembolic events. At the same time, components of the coagulation system, such as tissue factor (TF) and platelets, actively contribute to tumor progression. In particular, tumor cells often express high levels of TF, which allows them to evade the immune system and facilitate adhesion to the endothelium, angiogenesis, and subsequent metastasis [9]. Therefore, the generation of activated factors such as FXa and thrombin is not limited to thrombus formation. These molecules can activate receptors on tumor cells, known as protease-activated receptors (PARs), which stimulate cell growth, migration, and angiogenesis [10].

Factor XI (FXI), a 160 kDa glycoprotein serine protease, belongs to the intrinsic coagulation pathway [11]. Hemostasis is initiated when tissue factor (TF) or factor III (FIII) interacts with activated factor VII (FVIIa) in the TF pathway, with the subsequent formation of the TF–FVIIa complex. This interaction develops the conversion of factor X (FX) into its active form, activated factor X (FXa). FXa then initiates a series of events that culminate in the activation of factor II (FII), leading to the formation of fibrin and the production of a hemostatic clot [11]. FXI has a relatively minor role in hemostasis, as it consolidates the hemostatic clot through the activation of factor IX, after being activated primarily to FXIa by thrombin (FIX) [11].

Beyond clotting, factor XI might directly contribute to cancer growth. Possible mechanisms include tumor cell adhesion and migration [12], inflammation and immune evasion [13], and angiogenesis [12]. Our comprehensive review aims to define the relationships between factor XI and cancer and verify existing therapeutic opportunities targeting the factor.

## 2. Factor XI: Coagulation Cascade and Monitoring

The coagulation proteins are the core components of the coagulation system. They lead to a complex interplay of reactions that convert soluble fibrinogen to insoluble fibrin strands [14]. The cellular model of coagulation is a more modern and accurate view of the blood clotting process than the traditional cascade model. Unlike the cascade model, which separated the intrinsic and extrinsic pathways, the cellular model views coagulation as a process that occurs on cell surfaces and unfolds in three distinct but interconnected phases: initiation, amplification, and propagation [15]. The intrinsic pathway begins with the activation of factor XII (FXII) (a zymogen, an inactivated serine protease), which converts to activated factor XII (FXIIa) (an activated serine protease) upon exposure to subendothelial collagen [14]. The extrinsic pathway is the shorter pathway of secondary hemostasis. Once the vessel has been damaged, the endothelial cells release TF. The interaction of TF with FVII activates plasma FVII (FVII → FVIIa), with the subsequent formation of the TF–FVIIa complex. The TF–FVIIa complex activates FX [14].

**Figure 1:**
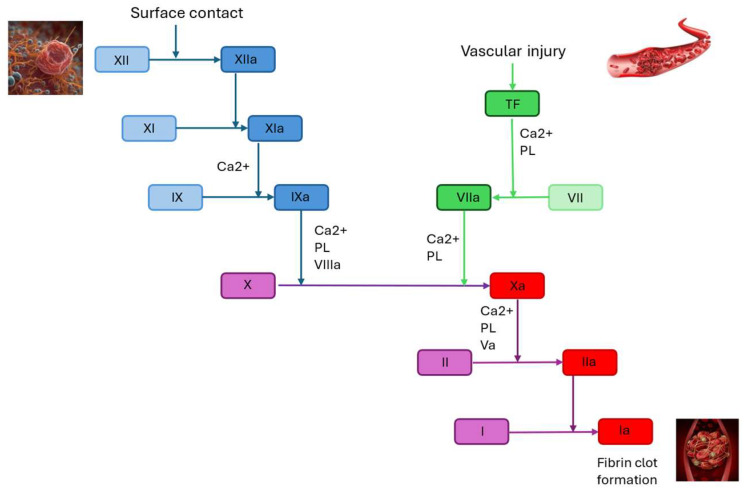
**Coagulation cascade.** PL: phospholipids; TF: tissue factor. Blue: intrinsic pathway; green: extrinsic pathway; red: common pathway. Inhibition of the common pathway is associated with a high risk of bleeding.

The common pathway commences at FX, which is activated to FXa. The process of activating FXa involves a complex reaction [14]. Figure 1 summarizes the coagulation cascade.

### 2.1. Role of Cancer in Thrombosis

Cancer and thrombosis are closely related. Cancer-associated venous thrombosis is a frequent and serious complication, second only to infection as a cause of death in cancer patients [16]. The relationship is bidirectional: cancer increases the risk of thrombosis, and thrombosis itself can influence tumor progression. First, cancer creates a state of hypercoagulability through several mechanisms: tumor cells can produce and release large amounts of TF, causing an increase in inflammatory cytokines, such as tumor necrosis factor-α (TNF-α) and interleukin (IL)-1β, and blood vessels can be directly damaged by therapeutic mechanisms, such as chemotherapy and radiotherapy [9]. The risk of thrombosis varies greatly among cancer patients. The main risk factors include the type and stage of cancer, as certain tumors, such as pancreatic, gastric, brain, and lung cancers, are associated with a particularly high risk [17]. Furthermore, the patient’s general condition, such as a high body mass index and advanced age, also play a role [17]. Cancer patients at high risk of thrombosis have elevated levels of biomarkers such as D-dimer, C-reactive protein (CRP), and TF, which are associated with increased risk. Measuring these markers, as in the Khorana score, can help stratify risk [18].

### 2.2. Role of Factor XI in Hemostasis and Thrombosis

FXI plays a relatively minor role in hemostasis, primarily activated to FXIa by thrombin. This process strengthens the final hemostatic clot by activating FIX. FXI is significant in pathological thrombus formation [19]. Pathological thrombosis can be initiated through two main pathways: the TF pathway and the contact pathway. In both cases, thrombin is generated during the initial phase, which activates FXI. In turn, FXI develops thrombus growth and propagation in an amplifying manner [20]. The essential role of FXI in thrombosis is supported by several animal studies, in which models deficient or inhibited in FXI exhibited a reduced incidence of thrombosis [21]. Furthermore, individuals with elevated levels of FXI were found to have a greater risk of VTE. In contrast, FXI deficiency is a rare, inherited bleeding disorder that can cause abnormal bleeding. It is also known as hemophilia C or Rosenthal syndrome. Unlike hemophilia A and B, which are X-linked, FXI deficiency is typically an autosomal recessive condition, meaning it affects both males and females equally [22]. Individuals with FXI deficiency experienced lower rates of ischemic stroke than the general population and infrequent spontaneous bleeding, suggesting that factor XI has a more critical role in thrombosis than in hemostasis [23]. A Mendelian randomization analysis by Gill et al. [24], including 16,169 patients with determined FXI levels, showed a causal effect of higher, genetically determined FXI levels on risk of any ischemic stroke (OR, 2.54; 95% CI, 1.68–3.84). At the same time, a prospective study by Rohmann et al. [25], including 576 patients, showed that high FXI activity was associated with a higher hazard of ischemic stroke (HR = 1.80, 95% CI 1.09–2.98). A retrospective study by Preis et al. [26], including 10,193 patients, of whom 8958 (88.9%) had normal factor XI activity, 690 (6.8%) had mild deficiency, and 542 (5.3%) had moderate–severe deficiency, showed that, compared to individuals with normal activity, the adjusted HR for cardiovascular events was 0.52 (95% CI, 0.31–0.87) in those with mild deficiency and 0.57 (95% CI, 0.35–0.93) in those with moderate–severe factor XI deficiency. The incidence of VTE was lower in those with factor XI deficiency compared to those with normal activity (adjusted HR = 0.26; 95% CI, 0.08–0.84).

Given the various roles of FXI in hemostasis and thrombosis discussed previously, inhibiting FXI has emerged as a promising strategy to separate the therapeutic benefits from the adverse effects of anticoagulant drugs [27]. In other words, targeting FXI may reduce the risk of thrombotic complications without increasing the likelihood of bleeding, which remains a significant concern with other types of anticoagulants.

## 3. Role of Factor XI in Cancer

### 3.1. Pathophysiology of Venous Thromboembolism

DVT is a pathological condition characterized by the formation of blood clots within the veins of the deep venous system. The term VTE refers to the association between DVT and PE. VTE represents the third most frequent cardiovascular disease in Western countries [28]. Historically, the incidence of VTE in Asian countries was lower compared to Western countries, but according to a recent systematic review by Lai Heng Lee et al., the incidence of VTE in Asian countries might be increasing [29]. In an observational cohort study by Cohen et al. [30], the incidence of first and recurrent VTE was analyzed. They considered 35,373 first VTE events (12,073 provoked, 16,708 unprovoked, and 6592 active cancer-associated VTE) among 26.9 million person-years. The overall incidence rate (IR) of VTE was 131.5 (95% confidence interval (CI), 130.2–132.9) per 100,000 person-years and 107.0 (95% CI, 105.8–108.2) after excluding cancer-associated VTE. The correlation between cancer and VTE has been well known since the time of Trousseau, who first identified this association [17]. Indeed, venous thromboembolism associated with cancer is a common complication among cancer patients, with an incidence significantly higher than that of the general population. In a large observational cohort study by Cohen et al. [30], the incidence of first and recurrent VTE in cancer patients was 5.8 (95% CI, 5.7–6.0) and 9.6 (95% CI, 8.8–10.4) per 100 person-years, respectively. VTE can lead to severe complications, such as post-thrombotic syndrome. VTE complications, which include post-thrombotic syndrome (PTS) and chronic thromboembolic pulmonary hypertension (CTEPH), are associated with substantial morbidity and high healthcare costs [31]. Fully understanding the mechanisms underlying thrombus formation and, thus, the development of VTE is essential to setting correct diagnostic and therapeutic pathways, which also prevent the development of the above-mentioned complications and recurrence of VTE. Historically, the pathophysiology of venous thrombosis is based on Virchow’s triad, which identifies three primary factors that contribute to thrombus formation in the veins: blood stasis, hypercoagulability, and endothelial injury. Blood stasis is often observed in situations such as prolonged immobility (for example, during a long flight or after surgery), in cardiovascular diseases like heart failure, or in cases of varicose veins. Hypercoagulability can be hereditary, as in the case of the FV Leiden mutation, prothrombin G20210A mutation, or antithrombin deficiency, or acquired, for example, due to the use of oral contraceptives, hormone replacement therapy, pregnancy, or certain types of cancer.

Endothelial or vessel wall injury plays a crucial role in the pathogenesis of venous thrombosis. It is characterized by an imbalance between procoagulant and anticoagulant substances and pro-inflammatory and anti-inflammatory mediators. This imbalance is often caused by increased oxidative stress, hypoxia, and reduced availability of nitric oxide. When the endothelial lining is damaged (for example, due to surgery, trauma, inflammation, or the use of medical devices such as catheters), underlying structures, including collagen, are exposed, which promotes platelet aggregation and activation of the coagulation cascade, thereby increasing the likelihood of thrombus formation. In cancer patients, all three components of the triad can be altered, thereby implicating them in the genesis of VTE. Indeed, in cancer patients, venous stasis is caused by immobility, hospital admission, perioperative status, or venous obstruction “ab estrinseco” by the tumor mass, which leads to a decrease in the clearance of activated coagulation factors. Regarding endothelial dysfunction, we know that von Willebrand factor (vWF), a marker of endothelial damage, is often elevated in patients with solid and/or hematological malignancies. Tumor cells release several pro-inflammatory and prothrombotic cytokines, such as TNF-α and L-1β, that can trigger prothrombotic processes.

Furthermore, an imbalance exists between procoagulant and anticoagulant factors. In particular, the levels of TF and fibrinogen are elevated, accompanied by a decrease in the levels of natural anticoagulants, such as antithrombin, protein C, and protein S. Virchow’s triad is, therefore, a fundamental concept in the pathophysiology of venous thrombosis, but it does not entirely encompass the entire picture. Thrombocytosis, characterized by the overactivation of platelets, is a common condition that contributes to the hypercoagulable state [32]. It is common for thrombus formation to be frequently triggered by endothelial damage and, thus, by the exposure of TF on damaged endothelial cells or its expression on the membrane of leukocytes [33]. As we know, the FVIIa/TF complex activates the coagulation cascade and thrombus formation, but the thrombus’s ability to propagate becomes limited once it extends beyond the damaged endothelial surface. In this context, FXI appears to be explicitly involved in thrombus growth [11]. Several preclinical studies support this observation [21,34]. Thrombosis in response to injury is attenuated in mice deficient in FXI, and the knockdown or inhibition of FXI reduces thrombosis in various animal models [21,34]. Individuals with FXI deficiency have a lower incidence of VTE and ischemic stroke compared to the general population. In contrast, those with high FXI levels have more than twice the risk of developing VTE [23,35].

**Figure 2:**
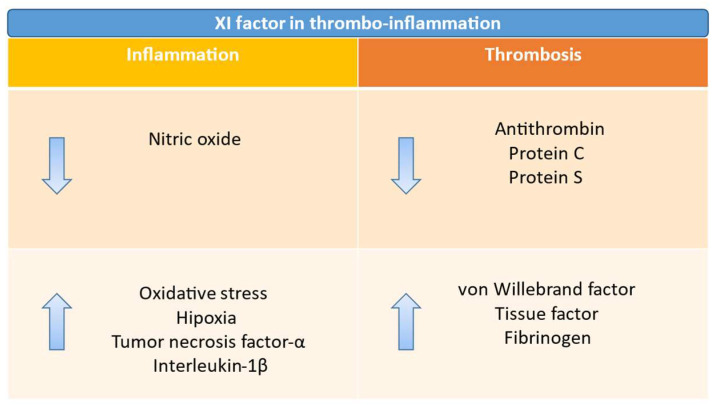
Factor XI in thrombo-inflammation. ↓: decrease; ↑: increase.

Interestingly, a Danish cohort study by Mulder et al. [36], including 499,092 patients with a first-time cancer diagnosis between 1997 and 2017 who were matched to 1,497,276 comparison individuals without cancer from the general population, found a rise of 6.5 times in the incidence of VTE in recent years among cancer patients. In contrast, it has remained essentially unchanged in the general population. This is likely due to the increased use of systemic therapies. It is well known that certain chemotherapeutic agents can promote the development of VTE. In the same cohort, a multivariate analysis revealed that immunotherapy, protein kinase inhibitors, and anti-angiogenic therapies are all risk factors that increase the risk of VTE. Figure 2 summarizes the role of FXI in thrombo-inflammation.

### 3.2. FactorXI and Cancer Progression

The close connection between inflammation and coagulation has long been well established, with the activation of one system capable of amplifying the response of the other, outlining a true crosstalk [37].

Specifically, although the direct relationship between these two systems is certain, the details and underlying pathways are still under investigation [38,39,40,41].

Several pieces of evidence and [13] research have emerged, aiming to understand this connection better and supporting a role for FXI that extends well beyond its involvement in coagulation.

In a scientific report by Robles et al. [13], 549 patients were evaluated, measuring factor XI coagulant activity (FXI:C) levels during an acute VTE event (*n* = 549) and 12 months later (*n* = 187), showing that FXI:C levels were significantly higher during the acute phase. In addition, in this investigation, they found several markers associated with FXI:C that have a role in T-cell activation and chemotaxis cluster of differentiation 28 (CD28) and chemokine ligand 16 (CCL16), inflammatory mediators (IL-1A, IL-18, and IL-27), and host defense proteins regenerating islet-derived protein 3-alpha and defensin alpha-1 (Reg3α). These FXI-related proteins were enriched in immune pathways related to causes of thrombo-inflammation, extracellular matrix interaction, lipid metabolism, and apoptosis.

**Figure 3:**
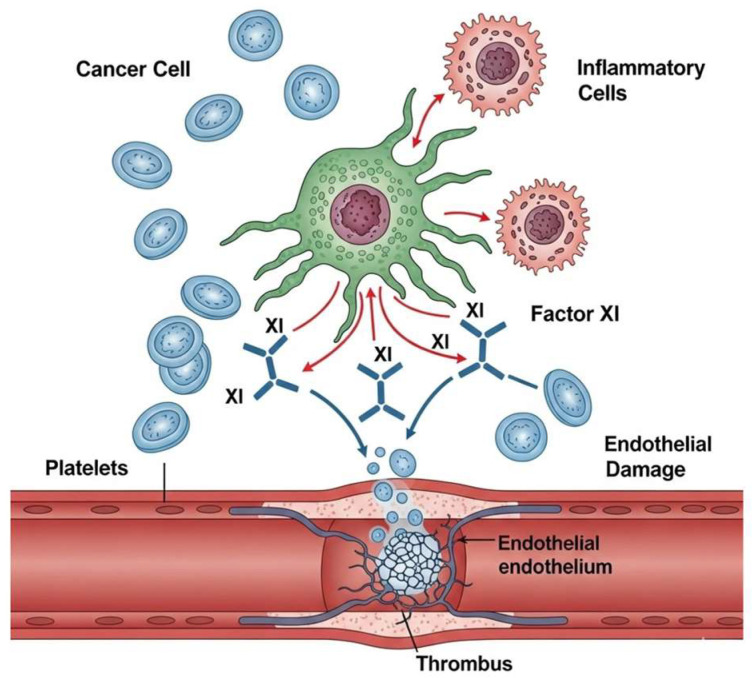
The role of factor XI in cancer.

Therefore, FXI plays a regulatory role in vascular permeability, but the mechanism is unknown. Puy et al. [12] reveal a novel pathway by which FXIa contributes to the development of inflammatory diseases, regulating vascular permeability. FXIa–plasminogen activator inhibitor-1 (PAI-1) complex binding to very low-density lipoprotein receptor (VLDLR) induces mitogen-activated protein kinase (MAPK) signaling, causing a disintegrin and metalloprotease (ADAM) 10-mediated cleavage of vascular endothelial cadherin (VE-cadherin). Achieving this by using small molecule antagonists that target FXIa and prevent FXIa–PAI-1 complex formation can alter inflammatory signaling and potentially mitigate the detrimental effects of excessive FXIa activation in cardiovascular diseases. Figure 3 represents the interaction between FXI and cancer.

In addition to a direct role in the inflammatory process, preclinical research has demonstrated that factors of coagulation are also active facilitators of cancer progression. However, there are not specific trials about the role of FXI. In Table 1, the preclinical and clinical trials that show the relationship between coagulant factors and cancer progression are summarized.

## 4. Factor XI Inhibitors

Many FXI inhibitors have been developed and are being studied. Several molecular forms have been developed, including small molecules (Asundexian and Milvexian) that bind to the active site of FXIa and inhibit its activity, antibodies directed against FXI that inhibit the activation of FXI or the activity of FXIa (Osocimab, Abelacimab, and Xisomab), antisense oligonucleotides (ASOs, e.g., Fesomersen) that reduce the synthesis of FXI by binding to the messenger ribonucleotide acid of FXI in hepatocytes and induce its degradation, and natural inhibitors, all synthesized in Table 1. Actually, there are several phase II and phase III studies ongoing about the use of FXI inhibitors in cancer patients to prevent or treat venous thromboembolic events, to prevent catheter-related thrombosis, and to prevent arterial thromboembolic events, summarized in Table 2.

### 4.1. FXI Inhibitors to Prevent Venous Thromboembolic Events

VTE remains a significant postoperative complication in patients undergoing major orthopedic surgery [55]. Prophylaxis of post-surgical VTE is one of the main clinical uses of conventional anticoagulants, such as low molecular weight heparin (LMWHs) and oral anticoagulants. For this reason, several phase II randomized trials have tested the safety and efficacy of FXI inhibitors in thromboembolic event prophylaxis for patients undergoing total knee arthroplasty.

Three randomized trials, summarized in Table 3, have evaluated FXI inhibitors for thromboprophylaxis following total knee arthroplasty [56,57,58,59].

These studies, however, excluded patients with active cancer; thus, their findings cannot be directly extrapolated to the setting of cancer-associated thrombosis. Patients with cancer have a 5–7-fold increased risk of VTE compared to the general population, with the risk further increased to 23-fold in patients receiving chemotherapy or targeted therapy [36,60]. Thrombosis causes hospitalization and is associated with significant morbidity in cancer patients [61]. In addition, VTE is the second leading cause of death in cancer patients [60]. Multiple studies [62,63,64] have confirmed that thromboprophylaxis lowers the incidence of thrombotic events in cancer patients receiving chemotherapy; however, this benefit is tempered by a concomitant increase in bleeding risk [65,66,67]. Khorana et al. [65] reported a significant increase in bleeding events among patients with a Khorana score ≥ 2 after 12 weeks of prophylactic dalteparin. Two studies evaluated FXa inhibitors in cancer patients: CASSINI and AVERT [66,67].

These findings highlight the ongoing clinical challenge of thromboprophylaxis in cancer-associated thrombosis. While thrombotic risk is markedly elevated in this population, bleeding risk is also significantly increased [68]. In this context, the development of clinical trials investigating FXI inhibitors may be of particular interest, as these agents appear to offer comparable efficacy to conventional anticoagulants for VTE prevention, with a potentially improved safety profile regarding bleeding.

### 4.2. FXI Inhibitors to Treat Venous Thromboembolic Events

Beyond thromboprophylaxis, the management of established VTE in cancer patients remains challenging, requiring clinicians to balance both the heightened thrombotic and bleeding risks inherent to this population. A meta-analysis by Frere et al. [69] incorporated six randomized controlled trials—Hokusai VTE Cancer, ADAM-VTE, SELECT-D, CASTA DIVA, CARAVAGGIO, and CANVAS [70,71,72,73,74,75]—including 3690 patients (male sex, 52%), aged a mean of 67 years, and comparing FXa inhibitors with LMWHs (1850 randomized to the DOACs arms and 1840 randomized to the LMWHs arms). The risk of recurrent VTE was significantly lower with DOACs compared to LMWHs (RR, 0.67; 95% CI, 0.52–0.85; *p* = 0.001), during a follow up of 3–6 months. The absolute risk reduction in VTE recurrence with DOACs was 2.7% (95% CI, –4 to –1.2; high certainty of evidence). However, their use was associated with a high incidence of clinically relevant bleeding. Patients on a DOAC regimen had a higher risk of major bleeding, although the difference did not reach statistical significance (RR, 1.17; 95% CI, 0.82–1.67; *p* = 0.39. The absolute risk increase with DOACs was 0.6% (95% CI, −0.7 to 2.5; high certainty of evidence), with a 3.7% risk of major bleeding in the LMWH group. Clinically relevant non-major bleeding was more frequent in patients treated with DOACs than in those treated with LMWH (RR, 1.66; 95% CI, 1.31 to 2.09; *p* < 0.0001). With a risk of clinically relevant non-major bleeding of 5.7% in patients receiving LMWHs, the absolute risk increase with DOACs was 3.8% (95% CI, 1.8–6.2). Building on the favorable outcomes of thromboprophylaxis in non-cancer populations, phase III trials were initiated to evaluate anticoagulant therapy in patients with cancer-associated thrombosis. In this setting, the competing risks of thrombosis and bleeding pose a significant clinical challenge, with all currently available agents demonstrating limitations in efficacy and safety. Importantly, bleeding risk appears to be influenced by tumor type, particularly in patients with unresected gastrointestinal or genitourinary malignancies [76]. In light of these considerations, two dedicated clinical phase 3 trials have been developed: ASTER (NCT05171049) and MAGNOLIA (NCT05171075).

ASTER (NCT05171049) [51] is a multicenter, randomized, open-label trial designed to compare abelacimab (administered once monthly) with apixaban (administered twice daily) for the treatment of cancer-associated VTE in approximately 1655 patients over a 6-month period. Primary outcomes include VTE recurrence, bleeding events, and treatment discontinuation at 6 months. MAGNOLIA (NCT05171075) [52] is a phase III, open-label study evaluating abelacimab versus daily subcutaneous dalteparin in approximately 1020 patients with VTE related to gastrointestinal or genitourinary malignancies. In both trials, abelacimab is administered subcutaneously at a dose of 150 mg once monthly following a single intravenous loading dose of 150 mg.

### 4.3. FXI Inhibitors to Prevent Catheter-Related Thrombosis

Despite the ubiquitous utilization of central venous catheters in clinical practice, their use commonly provokes thromboembolism [49]. In critically ill patients undergoing surgery, catheter-related thrombosis occurred in 37–64% of central venous catheters within 1 day of insertion and in 64–100% within 1 week [77]. FXI could represent a new target for device-associated thrombosis, which may reduce bleeding risk. Pfeffer et al. [49] enrolled 22 cancer outpatients undergoing central line placement to receive a single dose of gruticibart administered through the venous catheter within 24 h of placement. The incidence of catheter-associated thrombosis at day 14 was 12.5% in the 11 patients enrolled in the interventional study and 40.0% in the 11 patients in the parallel control study. These findings suggest that targeting FXI could be a safe intervention to prevent catheter thrombosis, but the small numbers and lack of a randomized control group limit the strength of any conclusions. NCT04465760 trial [78] was initiated but subsequently terminated due to the insufficient accrual rate. The study aimed to evaluate the role of Xisomab 3G3 in preventing catheter-related thrombosis in patients with cancer undergoing chemotherapy.

### 4.4. FXI Inhibitors to Prevent Arterial Thromboembolic Events

Emerging evidence suggests a mechanistic overlap between VTE and atherothrombosis [79], including in cancer patients [80,81]. Arterial thrombosis, like VTE, involves tissue factor-driven thrombin generation, which is amplified through factor XI and contributes to platelet activation. Despite the widespread use of antiplatelet agents in atherosclerotic disease, recurrent ischemic events remain common, indicating that platelet inhibition alone may be insufficient, especially in cancer patients, who may exhibit heightened thrombin activity. In the Cardiovascular Outcomes for People Using Anticoagulation Strategies (COMPASS) trial [82], 27,395 patients aged 68 ± 7.9 years, male 78%, with coronary artery disease or stable peripheral arterial disease were standed to one of three treatment arms: rivaroxaban 2.5 mg twice daily with 100 mg aspirin once daily; rivaroxaban 5 mg twice daily alone; or aspirin 100 mg once daily. The primary outcome, a composite of cardiovascular death, stroke, or non-fatal myocardial infarction, was significantly lower with rivaroxaban plus aspirin than with aspirin alone (4.1% and 5.4%, respectively; HR = 0.76; 95% CI, 0.66–0.86). However, the rate of major bleeding was significantly higher in the rivaroxaban plus aspirin group than in the aspirin-alone group. Among patients diagnosed with cancer during follow-up, bleeding events, such as gastrointestinal hemorrhage and hematuria, were strongly associated with new cancer diagnoses, raising safety concerns. These findings underscore the need for safer anticoagulant strategies in cancer populations, particularly agents like FXI inhibitors that may preserve efficacy while minimizing bleeding risk. To date, no trials have specifically evaluated arterial thrombotic risk or prevention strategies in patients with active malignancy.

In this context, an analysis from the AZALEA-TIMI 71 trial [83] compared bleeding outcomes between rivaroxaban and abelacimab in patients receiving concomitant antiplatelet therapy. The study demonstrated a statistically significant reduction in bleeding events among patients treated with abelacimab at doses of 90 mg or 150 mg plus antiplatelet agents compared with those receiving rivaroxaban plus antiplatelet therapy (HR, 0.26 and 0.30, respectively).

### 4.5. Stroke Prevention in Atrial Fibrillation

AF is the most prevalent cardiac arrhythmia [84], which affects approximately 1–2% of the general population, with prevalence rising markedly with age—approaching 10% in individuals over 75 years [85]. It is associated with a significantly increased risk of cardioembolic stroke and cognitive decline [86]. AF has been linked to a 39% elevated risk of developing cognitive dysfunction, often emerging several years to decades prior to clinical onset. This relationship remains significant even after excluding individuals with a prior history of stroke. Moreover, in the context of acute stroke, AF is associated with both earlier onset and a 2.7-fold higher risk of subsequent cognitive decline [87]. The current standard of care for stroke prevention in AF includes direct oral anticoagulants (DOACs), specifically FXa or FIIa inhibitors. However, pooled analyses from randomized clinical trials indicate annual rates of major bleeding around 4% and clinically relevant non-major bleeding near 10% among AF patients receiving DOAC therapy [88,89]. The encouraging results of early trials using FXI inhibitors for thromboprophylaxis in orthopedic surgery have generated increasing interest in extending this approach to patients with AF.

The potential benefit of factor XI inhibition in the long-term prevention of arterial thromboembolism—such as in AF—remains hypothetical, as clinical efficacy in reducing thrombotic events has yet to be demonstrated. The PACIFIC-AF trial [90], including 755 patients with AF, aged 73.7 years, 59% men, reported a lower incidence of bleeding with 20 mg or 50 mg of asundexian when compared to the placebo. Conversely, findings from the larger OCEANIC-AF trial [47], including 14810 patients, meanly aged 73.77 years, 64,8% men, showed an increased risk of stroke or systemic embolism with once-daily asundexian 50 mg relative to apixaban (hazard ratio, 3.79; 95% confidence interval [CI], 2.46 to 5.83). In contrast, the LIBREXIA-AF trial (NCT05757869) [48], a global phase III, randomized, double-blind trial including 15.500 patients with AF, uses milvexian at a twice-daily dosing regimen, which is a total daily dose four times higher than that of asundexian in OCEANIC-AF (NCT05643573). The arm of the trial is to evaluate the noninferiority of milvexian vs. apixaban.

The phase II AZALEA-TIMI 71 (NCT04755283) trial [50] was stopped early due to a significantly lower incidence of major and clinically relevant non-major bleeding in patients receiving monthly subcutaneous abelacimab (90 mg or 150 mg) compared with those on daily rivaroxaban (20 mg). Abelacimab causes suppression of free FXI levels, exceeding a 97% reduction from baseline at 3 months. The primary bleeding endpoint was reduced by 77% in the 90 mg group and by 67% in the 150 mg group, primarily driven by reductions in major, clinically relevant non-major, and gastrointestinal bleeding events. However, this study had an insufficient population to evaluate thrombotic efficacy, leaving doubts regarding the efficacy for antithrombotic protection provided by the doses tested. The ongoing phase III LILAC-TIMI 76 trial [91] is evaluating abelacimab 150 mg monthly versus placebo in about 1900 patients with AF who are ineligible for standard anticoagulation. This trial wants to assess the composite outcome of ischemic stroke or systemic embolism and will monitor bleeding events classified as Bleeding Academic Research Consortium (BARC) type 3c or 5.

Management of anticoagulant therapy in patients with AF and cancer remains particularly challenging, as these patients are at high thrombotic and hemorrhagic risk. The inclusion of patients with active cancer in the ongoing LILAC-TIMI 76 trial [91] represents an important step toward this clinical need. The results of this study are highly anticipated, as they could provide the possibility of using a new, safe, and effective drug in a high-risk subgroup historically excluded from most anticoagulant therapy trials. If successful, the management of these patients would change, offering the possibility of a paradigm shift in the prevention of cardioembolic events in vulnerable cancer patients with AF. Therefore, a subanalysis of the AZALEA–TIMI 71 showed reassuring data for the safety of abelacimab in patients undergoing invasive procedures during the duration of the study, but further studies are needed to evaluate this aspect [92]. Similarly, abelacimab appeared to reduce the risk of major bleeding in patients taking antiplatelet drugs when compared to rivaroxaban.

## 5. Limitations and Future Perspectives

This narrative review examines the pathophysiological link between FXI and cancer, highlighting current therapeutic opportunities. However, it is crucial to acknowledge the inherent limitations of the available studies that form the basis of this analysis. One of the main limitations is the heterogeneity of patient populations and the diversity of methodologies employed in clinical trials. Much of the evidence comes from observational and retrospective studies, which, while valuable, cannot establish definitive causality. Furthermore, most research has focused on a limited number of cancer types, leaving the complex interplay of FXI in other malignancies unexplored. The difficulty of isolating the specific role of FXI from other prothrombotic and protumor factors in a multifactorial setting such as oncology represents a further challenge.

Despite the limitations mentioned above, the growing interest in FXI as a therapeutic target opens up promising prospects. New FXI inhibitor molecules currently under development could overcome the limitations of traditional anticoagulants, offering greater safety and a reduced risk of bleeding. Large-scale, prospective, randomized, controlled trials will be crucial to validate preliminary results and define the precise role of these inhibitors in oncology clinical practice. Future research should also explore the impact of FXI modulation not only on the prevention of thromboembolic events but also on the progression of the cancer itself. The identification of specific biomarkers could enable a personalized medicine approach, identifying patients who would benefit most from factor XI-targeted therapy.

## 6. Conclusions

Thrombotic and bleeding complications are a frequent occurrence in cancer patients, increasing both comorbidity and mortality. Current therapeutic strategies have several shortcomings in this patient population, leading to the investigation of new molecular targets such as FXI. The use of FXI inhibitors appears to be a promising therapeutic strategy, offering potentially positive effects in the prevention and treatment of thromboembolic complications without significantly increasing the risk of bleeding, a limitation of conventional anticoagulants. New FXI inhibitor molecules currently under development could overcome the limitations of traditional anticoagulants, offering greater safety and a reduced risk of bleeding. The preliminary evidence is that further clinical trials are required and that the available data is not enough to make firm clinical recommendations. Several randomized clinical trials are ongoing to study FXI inhibitors in various clinical settings, aiming to demonstrate the efficacy and safety of FXI inhibition in cancer patients. Therefore, while FXI inhibition certainly represents a promising target in oncology today, the conclusion of these ongoing trials is necessary before these inhibitors can potentially be applied in clinical practice.

## Figures and Tables

**Table 1:** Preclinical and clinical trials that show the relationship between anticoagulant factors and cancer progression.

Trial	Population	Coagulation Factor	Inhibitor	Endpoints	Results
Preclinical [42]	Mouse model	Thrombin	Hirudin	Interaction of tumor cells with platelets and fibrinogen in isolated lung preparations	Tumor cell spreading and subsequent retention of the tumor cells in the lung were markedly inhibited in the anticoagulated mice
Preclinical [43]	Human cell lines	Tissue Factor	Anti-Tissue Factor monoclonal antibodies	The formation of platelet/fibrin/tumor cell aggregates may be causally related to endothelial adhesion and metastatic potential	Abolished prolonged adherence of metastatic cells in the vasculature and inhibited metastasis
Preclinical [44]	Human cell lines	Thrombin	R-hirudin and thrombin inhibitor peptides	The formation of platelet/fibrin/tumor cell aggregates may be causally related to endothelial adhesion and metastatic potential	To inhibit tumor progression, spread, and spontaneous metastasis
Clinical [45,46]		Factor XI	NA	In a prospective cohort of patients with NSCLC starting chemotherapy, contact system activation and thrombin generation biomarkers were assessed in relation to 6-month VTE occurrence and mortality	The 6-month VTE and mortality cumulative incidences were 11% and 27%, respectively. Basal levels of Factor XI activated: Antithrombin complexes were higher in patients who developed VTE than those in VTE-free patients

NSCLC: non-small cell lung cancer; VTE: venous thromboembolism.

**Table 2:** Factor XI inhibitors, mechanism of action, and clinical phase of experimentation.

Molecule	Mechanism of Action	Phase of Clinical Trial
Asundexian	bind to the active site of FXIa	OCEANIC-AF (NCT05643573) phase 3 [47]
Milvexian	bind to the active site of FXIa	LIBREXIA-AF trial (NCT05757869) phase 3 [48]
Xisomab	antibodies directed against FXI and FXII	(NCT04465760) Phase 2 [49]
Abelacimab	antibodies directed against FXI	AZALEA-TIMI 71 (NCT04755283) phase 2b [50] ASTER (NCT05171049) phase 3 [51] MAGNOLIA (NCT05171075) phase 3 [52]
Osocimab	antibodies directed against FXI	(NCT04523220) Phase 2b [53]
Fesomersen	antisense oligonucleotides	Phase 2b RE-THINC ESRD (NCT04534114) [54]

FXI: factor XI; FXII: factor XII; RE-THINC ESRD: Factor XI LICA to Reduce Thrombotic Events in End-Stage Renal Disease Patients on Hemodialysis.

**Table 3:** Clinical trials about the efficacy and safety of FXI inhibitors for thromboprophylaxis following total knee arthroplasty.

Trial	Agent	Phase of Clinical Study	Population (N)	Endpoints	Results
Active Comparator-Controlled Study to Assess Safety and Efficacy of ISIS-FXIRx in Total Knee Arthroplasty (FXI-ASO TKA) (NCT01713361) [56]	IONIS-FXI_Rx_	Phase II	300 patients	The primary efficacy outcome was the incidence of venous thromboembolism (assessed by mandatory bilateral venography or report of symptomatic events).	300 mg of IONIS-FXIRx significantly reduced VTE incidence (4%) compared to enoxaparin (30%) (*p* < 0.001), with similar bleeding rates (3% vs. 8%) (*p* < 0.001)
ANT-005 Total Knee Arthroplasty (TKA) trial [58]	Abelacimab	Phase II	412 patients	The primary efficacy outcome was venous thromboembolism, detected by mandatory venography of the leg involved in the operation or objective confirmation of symptomatic events.	Both 75 mg (*p* < 0.001) and 150 mg (*p* < 0.001) of abelacimab were more effective than enoxaparin in preventing VTE, without increasing bleeding risk; risk difference (95% CI) 1.9 (−0.7 to 4.5) and 0 for abelacimab 75 mg group and abelacimab 150 mg group, respectively
Antithrombotic treatment with Factor XIa inhibition to Optimize Management of Acute Thromboembolic events for Secondary Stroke Prevention (AXIOMATIC-TKA) trial (NCT03891524) [57]	Milvexian	Phase II	1242 patients	The primary efficacy outcome was venous thromboembolism (which was a composite of asymptomatic deep-vein thrombosis, confirmed symptomatic venous thromboembolism, or death from any cause)	Milvexian at a dose of at least 100 mg (administered once or twice daily) outperformed enoxaparin in VTE prevention (relative risk vs. enoxaparin 0.52; 0.42, 0.37, and 0.30), without a notable rise in bleeding risk (relative risks were 1.15, 1.14, 0.81, and 1.51)

## Abbreviations

The following abbreviations are used in this manuscript:

ADAM	a disintegrin and metalloprotease
AF	atrial fibrillation
AXIOMATIC-SSP	Antithrombotic treatment with Factor XIa inhibition to Optimize Management of Acute Thromboembolic events for Secondary Stroke Prevention
AVERT	A Very Early Rehabilitation Trial after stroke
BARC	Bleeding Academic Research Consortium
CAT	cancer-associated thrombosis
CCL 16	chemokine ligand 16
CD28	cluster of differentiation 28
CI	confidence interval
CTEPH	chronic thromboembolic pulmonary hypertension
DVP	deep-venous thrombosis
DOACs	direct oral anticoagulants
FII	factor II
FIII	factor III
FIX	factor IX
FOX-TROT	Fluoropyrimidine Oxaliplatin and Targeted Receptor Pre-Operative Therapy
FV	factor V
FVIIa	activated factor VII
FVIII	factor VIII
FX	factor X
FXI-ASO	Active Comparator-Controlled Study to Assess Safety and Efficacy of ISIS-FXIRx in Total Knee Arthroplasty
FXI	factor XI
FXII	factor XII
FXIIa	activated factor XII
FXa	activated factor X
GSDMD	gasdermin D
IL-1β	interleukin-1β
IR	incidence rate
LMWHs	low molecular weight heparin
LRR	leucine-rich repeats
MAPK	mitogen-activated protein kinase
NACHT	nucleotide-binding domain function
NLRP3	pyrin domain–containing protein 3
PAI-1	plasminogen activator inhibitor-1
PE	pulmonary embolism
PT	prothrombin time
PTS	post-thrombotic syndrome
PTT	partial thromboplastin time
TF	tissue factor
TNF-α	tumor necrosis factor-α
VE-cadherin	vascular endothelial cadherin
VLDLR	very low–density lipoprotein receptor
VTE	venous thromboembolism
vWF	von Willebrand factor
